# Fate of Pyriproxyfen in Soils and Plants

**DOI:** 10.3390/toxics8010020

**Published:** 2020-03-13

**Authors:** James Devillers

**Affiliations:** CTIS, 3 Chemin de la Gravière, 69140 Rillieux-La-Pape, France; j.devillers@ctis.fr

**Keywords:** pyriproxyfen, soil, plant, metabolites, insect growth regulator, endocrine disruptor, terrestrial ecosystems

## Abstract

Since the 1990s, the insect growth regulator pyriproxyfen has been widely used worldwide as a larvicide in vector control and in agriculture to fight a very large number of pests. Due to its widespread use it is of first importance to know how pyriproxyfen behaves in the terrestrial ecosystems. This was the goal of this work to establish the fate profile of pyriproxyfen in soils and plants. Thus, in soil, pyriproxyfen photodegrades slowly but its aerobic degradation is fast. The insecticide presents a high tendency to adsorb onto soils and it is not subject to leaching into groundwater. On the contrary its two main metabolites (4′-OH-Pyr and PYPAC) show a different fate in soil. When sprayed to plants, pyriproxyfen behaves as a translaminar insecticide. Its half-life in plants ranges from less than one week to about three weeks. The review ends by showing how the fate profile of pyriproxyfen in soils and plants influences the adverse effects of the molecule on non-target organisms.

## 1. Introduction

The behavior of pesticides in soils is under the dependence of physical, chemical, and biological processes including volatilization, sorption–desorption, aerobic and anaerobic degradation, uptake and release by plants, run-off, and leaching. These various complex processes control the transport of pesticides within the soil and their transfer from the soil to the other environmental compartments [1,2,3]. The relative importance of these processes varies with the structure and physicochemical properties of the pesticide, the soil characteristics, the climatic conditions, and the plant-soil system considered.

Pyriproxyfen (4-phenoxyphenyl (*RS*)-2-(2-pyridyloxy) propyl ether) is a broad spectrum insect growth regulator mimicking the functioning of the juvenile hormone. It disrupts the hormonal system of the insects interfering with their development and transformation into adults [4]. Pyriproxyfen is used for controlling household pests such as the German cockroach (*Blattella germanica*) [5] and the common housefly (*Musca domestica*) [6] as well as for prophylactic flea control on cats and dogs [7,8]. Pyriproxyfen is particularly efficacious as larvicide against all the mosquito species [9,10,11]. It allows us to fight pests on a large number of agricultural plants depending on the regulation of the country in which it is used. These plants include apple, almond, apricot, banana, bean, cabbage, carrot, citrus, cotton, cucumber, grape-wine, mango, melon, olive, papaya, pear, pepper, soybean, strawberry, tomato, watermelon, and zucchini [12,13,14,15,16,17,18]. The study of the behavior of pyriproxyfen for a given crop in the growing conditions of a specific area is compulsory to estimate whether the established pre-harvest time ensures that the residue levels are below the maximum residue level (MRL) that is the highest concentration of pesticide that is legally tolerated in or on food when it is applied under good agricultural practices. Thus, recently, Efsa [19] proposed 0.4 mg/kg as MRL for the tomato and 0.7 mg/kg for the citrus.

Due to the widespread use of pyriproxyfen worldwide, it is of first importance to know how this insecticide behaves in the terrestrial environment. As a result, the goal of this study was to establish the fate profile of pyriproxyfen in soils and plants. This will provide better insights into the processes of contamination of the various terrestrial ecosystems by this insecticide. In addition, the bioavailability of pyriproxyfen, namely the amount of the insecticide that is freely available to the living organisms of these ecosystems and that can lead to adverse physiological or toxicological responses will be better assessed.

## 2. Fate in Soils

### 2.1. Photolysis

**Figure 1:**
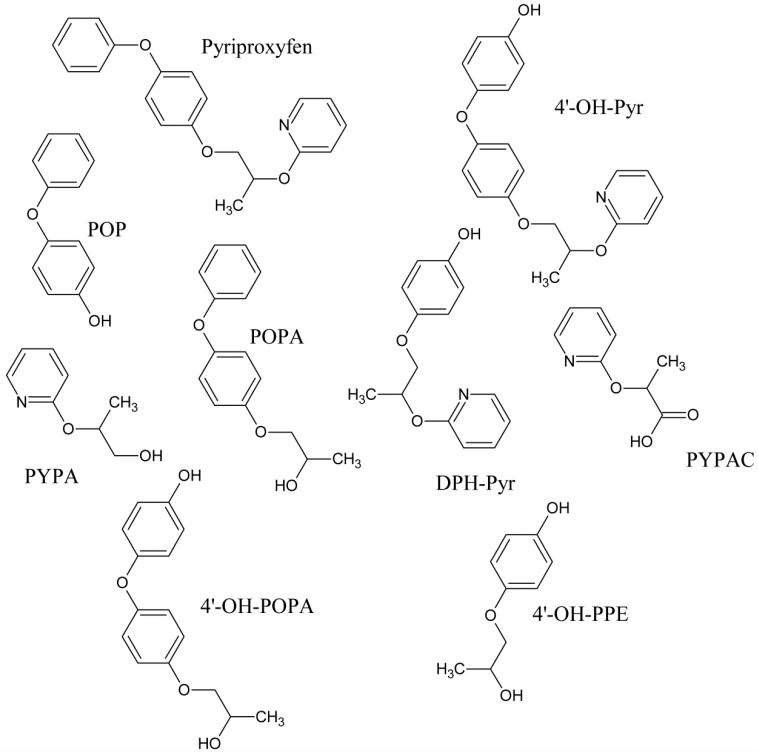
Pyriproxyfen and its main degradation products in soils and plants.

Photodegradation of radiolabeled pyriproxyfen was evaluated on sandy loam and silty loam soils in natural sunlight for eight weeks. In the former soil, the half-lives of the phenyl and pyridyl labels were equal to 12.5 and 10.3 weeks while in the silty loam soil, they were of 18 and 21 weeks, respectively [20]. Photodegradation was also studied in sandy loam soil (~0.3 mg/kg) with artificial sunlight (xenon lamp) for 20 and 18 days (12 h irradiation per day). The half-lives of the phenyl and pyridyl labels were respectively equal to 16 and 6.8 days in irradiated samples and 27 and 13 days in dark control samples [21]. As regards the pyridyl label, (*RS*)-2-(2-pyridyloxy)propionic acid (PYPAC, Figure 1) was identified as the main degradation product reaching its maximum level on day 10. 4-(4-hydroxyphenoxy) phenyl (*RS*)-2-(2-pyridyloxy)propyl ether (4′-OH-Pyr, Figure 1) was also identified from day 14 and 4-hydroxyphenyl (*RS*)-2-(2-pyridyloxy)propyl ether (DPH-Pyr, Figure 1) only at day 18. As regards the phenyl label, 4′-OH-Pyr reached its maximum level at day 10 [21]. Fenoll et al. [22] studied the effects of soil solarization (SO) and biosolarization (SB, as well as solarization and biofumigation) on the dissipation of pyriproxyfen. During the summer season in Murcia (Spain), 17-L pots were filled with clay-loam soil (33% clay, 30% silt, 37% sand, pH = 7.86, 1.59% organic matter content (OM), and electrical conductivity = 3.54 dS m^–1^). The organic matter used for SB was a mixture of sheep and chicken manures (63.1%). For SO and SB experiments, pots were covered with a low-density polyethylene film (50 µm thick). In addition, manure was applied to the SB pots (400 g/pot). Control pots (not exposed) and those submitted to SO and SB were spiked with pyriproxyfen (Juvinal commercial product, 10% emulsifiable concentrate (EC)). Sampling and analysis were made up to 90 days after the beginning of the experiments. The DT_50_ (time required for dissipating 50% of the initial concentration) values found for the control, the SO and SB experiments were equal to 81, 39, and 13 days, respectively. The high rate of organic matter in SB enhances the degradation rate of pyriproxyfen. In practice this implies that organic matter application via biosolarization can enhance the bioremediation effect of solarization against pyriproxyfen [22].

### 2.2. Metabolism

Radiolabeled pyriproxyfen was used to evaluate its persistence in a sandy clay loam soil (73% sand, 8% silt, 19% clay, 2.4% OM, pH = 5.7) at 0.5 mg/kg under aerobic conditions at 25 °C in the dark for 30 days. Pyriproxyfen disappeared rapidly the first days and then more slowly leading to an estimated half-life of 28 days after the first seven days. The estimated mineralization half-lives equaled 68 and 139 days for the pyridyl and phenyl labels, respectively. The main identified residue was 4′-OH-Pyr and the minor products were PYPAC, DPH-Pyr and 4-(4-hydroxyphenoxy)phenyl (*RS*)-2-hydroxypropyl ether (4′-OH-POPA, Figure 1) [21]. The same experiment was performed in a sandy loam soil (54% sand, 36% silt, 10% clay, 0.8% OM, pH = 6.5) but incubated for 91 days. Again, a two disappearing step was observed leading to estimated half-lives of 8.2 days during days 1–14 and 20 days during days 14–91. The estimated mineralization half-lives were 112 and 82 days for the phenyl and pyridyl labels, respectively. PYPAC and 4′-OH-Pyr were the main and minor identified products, respectively [21].

[Pyr-^14^C]- and [Phe-^14^C]-radiolabeled pyriproxyfen (RP) were incubated aerobically in a sandy loam soil (8.4% clay, 0.87% OM, pH = 7.6) at 0.6 mg/kg in the dark at 25 °C for six months. Mineralization was slow with, respectively, 13.9% and 31.1% of the ^14^C from the phenyl and pyridyl labels that evolved as CO_2_ after 180 and 189 days [21]. This was equivalent to mineralization half-lives of 850 and 330 days. Pyriproxyfen disappeared rapidly in the first 14 days with estimated initial half-lives of 6.3 and 9.1 days for the phenyl and pyridyl labels, but much more slowly after, with respectively estimated half-lives of 120 and 77 days between two and six months. 4′-OH-Pyr was the main degradation product for [Phe-^14^C]-RP. It was detected at day 7, reached the highest measured level on day 14 and then decreased. DPH-Pyr was a minor product detected at day 14 and which decreased to be found at <0.1% at day 180. As regards, [Pyr-^14^C]-RP, 4′-OH-Pyr, PYPAC, and DPH-Pyr (minor) also showed a maximum level at day 14 and then decreased.

The polar material, which accounted for 23% of the applied ^14^C after 180 days in the case of [Phe-^14^C]-RP and 22% after 189 days with [Pyr-^14^C]-RP, could not be identified but was characterized as a mixture of high-molecular-weight compounds, suggesting incorporation or binding to natural products [21]. It is assumed that the aerobic soil metabolism of pyriproxyfen follows first-order kinetics. In an aerobic soil, Sullivan and Goh [20] estimated, respectively, half-lives of 12.4, 8.17, and 0.69 days for pyriproxyfen, PYPAC, and 4′-OH-Pyr but the characteristics of the soil were not given.

About 30% of the currently registered pesticides contain one or more chiral centers [23]. This may lead to different environmental fate behaviors and biological activities for their enantiomers [24,25,26].

In this context, Liu et al. [27] evaluated the aerobic biodegradation of the two pyriproxyfen enantiomers in five types of soils and one sand site. The different half-lives found are listed in Table 1 with the main characteristics of the media.

Inspection of Table 1 shows that in sand, the degradation is much slower than in the five soil types in which the organic matter content seems to be the critical factor for the degradation of pyriproxyfen. Enantioselective degradation is observed in sandy loam soil and in sand. In both cases, (+)-pyriproxyfen degrades faster than (-)-pyriproxyfen. Faster degradation is also observed when the moisture is at 25% rather than 10% and 50%. It seems that the presence of imadacloprid with pyriproxyfen slightly accelerates the degradation of the juvenoid while the mixture with emamectin benzoate can drastically change the persistence of pyriproxyfen. This is the case for the loam soil type (Table 1). Last, a second application of pyriproxyfen slows down the degradation of the juvenile hormone mimic in the sandy loam soil. Indeed, the t_1/2_ values of (+)-pyriproxyfen are equal to 4.82 days and 11.99 days after the first and second treatment, respectively. Those of the (-)-pyriproxyfen are equal to 5.62 days and 14.68 days, respectively.

The dissipation of pyriproxyfen in soil also depends on the concentration used. Chang et al. [28] showed that the half-lives of pyriproxyfen at 1, 5, and 10 mg/kg at 25 °C in a clay loam soil (28.5% sand, 31% clay, 40.5% silt, pH = 5.5) were equal to 4.80, 43.74, and 48.27 days, respectively. They also showed that the t_1/2_ values decreased with an increase in the temperature. Thus, at 10 mg/kg, t_1/2_ values were equal to 48.27, 36.93, and 34.39 days at 25, 30, and 40 °C, respectively.

Liu et al. [29] showed that the enantioselective biodegradation of pyriproxyfen in soil changed in presence of fertilizers. While the t_1/2_ values of (-)-pyriproxyfen and (+)-pyriproxyfen in the control sandy clay loam soil (63.2% sand, 12% silt, 24.8% clay, 7.57% OM, pH = 8.49) equaled 16.67 days and 16.91 days, in the presence of compound fertilizer, the t_1/2_ values dropped to 9.88 days and 9.58 days, respectively. On contrary, addition of either organic fertilizer or potassium dihydrogen phosphate slowed down the dissipation of pyriproxyfen enantiomers with t_1/2_ values of 20.07 days/20.46 days and 20.29 days/18.76 days for the (-)-pyriproxyfen/(+)-pyriproxyfen, respectively. Last, Liu et al. [29] showed that pyriproxyfen did not dissipate after using urea for 42 days. Liu et al. [30] also investigated the enantioselective biodegradation of the metabolites of pyriproxyfen in five different soils. The characteristics of the soils are summarized in Table 2 with the biodegradation results.

Liu et al. [30] found that pyriproxyfen mainly degraded to 4′-OH-Pyr and DPH-Pyr but only the kinetics of the former metabolite was clearly observed with the five studied soils. Indeed, DPH-Pyr was only quantified in two soils with low concentrations. Inspection of Table 2 shows that the fastest dissipation of pyriproxyfen occurred in the loamy sand soil and the slowest in the clay #2 soil. No enantioselectivity in degradation was observed for these two soils. On contrary slight differences were found for the 4′-OH-Pyr. However, while in the loamy sand soil (+)-4′-OH-Pyr degraded faster than (-)-4′-OH-Pyr in the clay #2 soil, this was the converse. Enantioselectivity was observed in the clay #1 and sandy clay loam #1 soils both for pyriproxyfen and 4′-OH-Pyr. Although the t_1/2_ values of (-)-Pyr and (+)-Pyr ranged from 2.11 days to 9.61 days and from 2.11 days to 9.69 days in the five soils, those of (-)-4′-OH-Pyr and (+)-4′-OH-Pyr ranged from 3.29 days to 11.21 days and from 2.8 days to 13.30 days, respectively (Table 2). Liu et al. [30] demonstrated that soil enzyme activity was perturbed by the presence of pyriproxyfen. Soil sucrase activity (in mg of reducing sugar produced per g of soil sample per day) decreased from 31.80 ± 1.14 mg/day/g to 16.37 ± 0.33 mg/day/g at 60 days followed by a reduction to 9.49 ± 0.12 mg/day/g at 120 days in the sandy clay loam soil #1. The same trend was observed in the loamy sand soil. Sucrase was not sensitive to pyriproxyfen within 120 days in clay soils #1 and #2 and in the sandy clay loam soil #2. On contrary, catalase activity (in µmol H_2_O_2_ degraded per g of dry soil sample per day) increased with time in the loamy sand soil and in the sandy clay loam soil #1. Thus, for example, as regards the latter soil, the catalase activity after 0, 60, and 120 days of incubation equaled 32.48 ± 2.6, 41.04 ± 2.96, and 50.45 ± 0.53 µmol/day/g, respectively. The catalase activity increased from 18.52 ± 2.82 µmol/day/g to 23.02 ± 0.54 µmol/day/g at 60 days and then significantly decreased to 13.02 ± 0.15 µmol/day/g at 120 days in the clay soil #1. While, the short decrease of catalase activity at 60 days in the sandy clay loam #2 soil was not significant (10.92 ± 0.40 µmol/day/g versus 9.34 ± 0.64 µmol/day/g) a clear restauration was observed at 120 days (12.98 ± 0.78 µmol/day/g). Activity of urease (in µg NH_3_-N generated per g of soil sample per day) increased in the sandy clay loam #1 soil from initial 150.07 ± 9.93 µg/day/g to 291.95 ± 61.36 µg/day/g at 60 days and then to 791 ± 147.52 µg/day/g at 120 days. The same trend was observed for the sandy clay loam soil #2. Although a decrease in urease activity was observed in the loamy sand soil from 698.06 ± 9.85 µg/day/g to 504.62 ± 97.81 µg/day/g at 60 days, a significant increase to 746.72 ± 92.04 µg/day/g was observed at 120 days. This trend was also observed in the clay soil #2. On the contrary, in clay soil #1, a strong increase in urease activity was observed at day 60 followed by also a strong decrease at day 120. A decrease in dehydrogenase activity (in µg of triphenyl formazone produced per g of soil sample per day) was observed in loamy sand soil with 37.72 ± 2.18 µg/g/day, 29.59 ± 1.73 µg/g/day, and 16.81 ± 4.33 µg/g/day at days 0, 60, and 120, respectively. The same trend was observed in the clay soils #1 and #2. In the sandy clay loam soil #1, the dehydrogenase activity decreased from 95.07 ± 1.12 µg/g/day to 24.49 ± 1.23 µg/g/day at 60 days and then increased to 35.11 ± 3.68 µg/g/day at 120 days. On contrary, in the sandy clay loam soil #2 the dehydrogenase activity increased from 19.26 ± 1.98 µg/g/day to 30.04 ± 5.88 µg/g/day at 60 days and then decreased to 16.64 ± 1.23 µg/g/day at 120 days [30].

### 2.3. Mobility

Leaching is the vertical displacement of contaminants, such as water-soluble pesticides or fertilizers, carried by water downward through the soil profiles to the groundwater [31,32]. Soil texture and structure, organic matter and water content affect the leaching of pesticides in soil. The physicochemical properties of the pesticides also influence the leaching behavior of pesticides [33,34]. Koc, which is defined from the distribution coefficient (kd) and the organic carbon fraction of the soil, is the key parameter for measuring the relative mobility of pesticides in soils. The parameter has been related to the 1-octanol/water partition coefficient (log Koc = f (log Kow)) [35]. Pesticides with high Koc values are prone to a lower mobility in soils than those with low Koc values. Fenoll et al. [36] established such a relationship with pyriproxyfen. From a laboratory leaching study performed on a clay loam soil, they showed that the pyriproxyfen was not a leacher. In another leaching study with fine sandy loam, loamy sand, clay, and loam soils, Schaefer et al. [37] showed that over 50% of the pyriproxyfen applied remained in the upper 6 cm of the 30-cm soil columns, demonstrating that pyriproxyfen did not have capacity for downward migration.

[Phe-^14^C]-RP was mixed at 1 mg/kg to silt soil (43% sand, 47% silt, 10% clay, 7.6% OM, pH = 7) and to a sandy loam soil (72% sand, 17% silt, 11% clay, 0.9% OM, pH = 7.2) in columns that were leached with 360 mL of water at 69 mm/day for eight days [21]. Most of the ^14^C (89% for the silt soil and 84% for the sandy loam soil) still remained in the treated portion at the top of the columns, with 5% in the 0–5 cm untreated layer of both soils. Small amounts of ^14^C were detected throughout the columns with 0.1% and 2.8% in the eluates from the silt and sandy loam, respectively. Most of the extractable residue was pyriproxyfen with bound ^14^C in the humin, humic acid, and fulvic acid fractions. Small amounts of 4′-OH-Pyr and DPH-Pyr were identified in the extracted and fulvic acid fractions. In this study, pyriproxyfen also appeared unlikely to be a leacher and its degradation products were substantially bound in the soil organic matter [21].

In another study, [Phe-^14^C]-RP and [Pyr-^14^C]-RP were incubated with a sandy loam soil (23% clay, 0.79% OM, pH = 8.1) under aerobic conditions at 0.6 mg/kg in the dark at 25°C for 14 days while a leaching study was initiated at day 9. In the experiment with [Phe-^14^C]-RP, 86% of the ^14^C remained in the treated soil (top 3 cm), with 2.3% in the 3–9 cm portion, 0.13–0.54% in the other portions and 1% in the leachate giving a total recovery of 90%. 4′-OH-Pyr and DPH-Pyr were again identified. With [Pyr-^14^C]-RP, 88.5% of the ^14^C remained in the top 3 cm, 1.4% in the 3–9 cm section, 0.13–0.44% in the other sections and 7.6% in the leachate. The total recovery was of 98.6%. Most of the ^14^C was associated to pyriproxyfen (35%) and bound residue (41%). In addition to 4′-OH-Pyr and DPH-Pyr, PYPAC was also identified representing the main residue in the leachate. It was found apparently mobile [21].

Pyriproxyfen presents high soil adsorption coefficients (Table 3) and is strongly adsorbed to most agricultural soils.

This has to be related to the low water solubility of pyriproxyfen which is only equal to 0.367 ± 0.004 mg L^−1^ at 25 °C and pH = 6 and to its high *1*-octanol/water partition coefficient, log Kow of 5.37 at 25 °C and pH = 5.6 [38].

With a solubility value in water of 1.1 mg/L at 25 °C, 4′-OH-Pyr adsorbs less on soils than pyriproxyfen (Table 4) and it has a slight chance of leaching. On contrary, PYPAC, with a water solubility of 89.1 g/L at 21.4 °C [21] presents low Koc values (Table 4). It is prone to have a high or very high mobility in the soils with a high potential to leach to groundwater.

## 3. Fate in Plants

### 3.1. Identification of Metabolites

Radiolabeled pyriproxyfen was topically applied to tomato fruits (*Lycopersicon esculentum* Mill. cv. Ponterosa) in acetonitrile or emulsifiable solutions at a rate of 60 g a.i./acre (about 150 g/ha) at 35, 21, and seven days before harvest [39]. The acetonitrile solution was used for investigating the potential metabolism of pyriproxyfen while the emulsifiable concentrate was employed for assessing metabolic profiles under agriculture practices. Application of the former solution showed that 73.65% ([Phe-^14^C]-RP) and 60.15% ([Pyr-^14^C]-RP) of total radioactive residues were present in the surface rinse fraction. The radioactivity recovered from the rinse was all the unaltered pyriproxyfen. Metabolites were found in juice and pomace extracts. In the treatment with [Phe-^14^C]-RP, POP (0.02% of total radioactive residues) was found as a free metabolite and 4′-OH-POPA (0.06%) and 4-hydroxyphenyl (*RS*)-2-hydroxypropyl ether (4′-OH-PPE, 0.05%) (Figure 1) were found as conjugates in the juice fraction. 4′-OH-Pyr (0.28%), (*RS*)-2-hydroxypropyl 4-phenoxyphenyl ether (POPA, 0.13%), and 4′-OH-POPA (0.01%) were detected as free metabolites from the extract of pomace. DPH-Pyr was a common metabolite of [Phe-^14^C]-RP and [Pyr-^14^C]-RP but it was only detected in the pomace extract of the latter (0.14%). In addition, PYPAC (0.18%), (*RS*)-2-(2-pyridyloxy) propanol (PYPA, 0.08%), and 4′OH-Pyr (1.06%) were found in the pulpy residue. Use of the emulsifiable concentrate solution highly reduced the radioactive residues on the fruit surface since only 12.40% ([Phe-^14^C]-RP) and 13.46% ([Pyr-^14^C]-RP) of total radioactive residues were present in the surface rinse fraction. 4′-OH-Pyr was also found in surface with 0.06% and 0.02% of total radioactive residues with [Phe-^14^C]-RP and [Pyr-^14^C]-RP, respectively. The metabolite was also found in the pomace extract with 1.03% and 1.13%, respectively. POPA (0.10%) and POP (0.03%) were also observed as free metabolites from the pomace of [Phe-^14^C]-RP. It is worthy to note that these two metabolites were not detected from the pomace of [Pyr-^14^C]-RP. Trace of 4′-OH-PPE was released by acidic hydrolysis of the juice fraction of [Phe-^14^C]-RP. On contrary, no trace amount of known metabolites were detected from the juice of [Pyr-^14^C]-RP. According to Fukushima et al. [39], pyriproxyfen primarily undergoes hydroxylation at the 4′-position of the terminal phenoxyphenyl ring to form 4′-OH-Pyr, cleavage of the propylpyridyl ether to form POPA, cleavage at the propyl phenyl ether to form PYPA and POP, desphenylation, following conjugation of these metabolites.

Apple trees were treated soon after petal fall and at 60 and 40 days before harvest with [Phe-^14^C]-RP and [Pyr-^14^C]-RP at 150 g/ha [21]. Surface wash with acetonitrile removed 1.5–2.6% of total residue. Washed apples were homogenized and centrifuged to obtain juice and pomace that contained 6–14% and 84–92% of the ^14^C, respectively. Pyriproxyfen was not detected in the juice, where the main identified residue was PYPA (25% of the ^14^C) but accounted for most of the radioactivity in the pomace [21].

Field cotton plants were treated 43 and 28 days before harvest with [Phe-^14^C]-RP and [Pyr-^14^C]-RP at 150 g/ha. Pyriproxyfen was the main residue component found in the gin trash. Residues were much lower in the seed than in the gin trash, suggesting a very limited (if any) translocation of residue from leaf to seed. Pyriproxyfen constituted only 3.9% ([Phe-^14^C]-RP) and 0.6% ([Pyr-^14^C]-RP) of the residue in the seed, where the main identified residue was PYPAC in free and conjugated forms. Much of the residue in the cotton seed (49% [Phe-^14^C]-RP and 55% [Pyr-^14^C]-RP) was non-extractable; about half of this was associated with the protein, carbohydrate, and lignin fractions [21].

### 3.2. Dissipation

Dissipation of a chemical after its application on plants depends on numerous factors including the plant species and its developmental stage, the physicochemical properties of the chemical and its formulation, the application method, and the climatic conditions (wind, humidity, temperature, etc.).

Sulaiman et al. [40] studied the dissipation of pyriproxyfen after application on tomatoes (Admiral commercial product, 10% EC at 0.75 mL/L) growing in greenhouses. The results showed that the initial deposit of pyriproxyfen equaled 2.89 ± 0.25 mg/kg. After 1, 3, 5, 7, 9, and 14 days, the losses of pyriproxyfen were of 18.34%, 36.21%, 42.07%, 52.07%, 70.59%, and 84.08%, respectively. The half-life (t_1/2_) was estimated to 5.41 days.

Fenoll et al. [41] investigated the dissipation rate of pyriproxyfen in pepper (*Capsicum annuum* L., cv. Almuden) growing in a greenhouse of 600 m^2^. Treatments were applied with a sprayer using the Atominal commercial product (10% EC pyriproxyfen at 0.75 mL/L). Two applications (60 g a.i./ha) were made 15 days apart. Samples were collected two hours after application I and then after 1, 3, 7, and 15 days. A same sampling strategy was applied after application II except that a sample was also collected at day 21. The relative humidity during applications I and II was 51% and 60% with a temperature of 23 °C and 19 °C, respectively. After the first application, pyriproxyfen showed a very low residue (0.12 mg/kg) that was due to the low application rate. However, the degradation rate was rather low. The decay rate showed a pseudo-first-order kinetics and a t_1/2_ of 21.47 days. For comparison purposes, it is interesting to note that pirimicarb that was also investigated in this study (400 g a.i./ha) presented a t_1/2_ value of 4.41 days after the first application. After the second application, the residue level of pyriproxyfen was 0.24 mg/kg and the t_1/2_ value calculated with pseudo-first-order kinetics was 18.57 days [41].

After an application of Admiral, 10% EC at 0.75 mL/L on green pepper fruits, Sulaiman et al. [40] found 6.71 ± 1.73 mg/kg of pyriproxyfen. After 1, 3, 5, 7, 9, and 14 days, decreases of 20.27%, 31.30%, 43.96%, 49.48%, 64.83%, and 88.08% were observed, respectively. A t_1/2_ value of 5.41 days was found. Due to the lack of information on the experimental conditions, it is difficult to make a comparison with the results found by Fenoll et al. [41] after the first application of pyriproxyfen.

Dong et al. [42] evaluated the dissipation and residue levels of pyriproxyfen (8% suspension concentrate (SC)) in Chinese citrus named Shan xia hong. During the field test, the temperatures ranged from 4 °C to 38 °C, and the accumulative rainfall was about 1370 mm. Two hours after application, 0.14 mg/kg of pyriproxyfen was found in the citrus samples. The t_1/2_ value was estimated to 13.3 days. The residues in citrus peel ranged from 0.063 to 0.37 mg/kg. Those in citrus pulp were lower being below the LOQ of 0.005 mg/kg.

Paya et al. [43] studied the dissipation of pyriproxyfen in peach fruit (*Prunus persica* cv. catherine) in an experimental field of a producer farm. Two field dissipation studies were carried out, one at the pre-harvest interval (i.e., time between the last pesticide application and crop harvesting, PHI) with good agricultural practice (GAP) in the use of the treatments and the other one in a critical agricultural practice (CAP) situation. A backpack sprayer with a pocket pistol was used to apply the Atominal (10% EC pyriproxyfen) on the peach trees. It was applied a volume of 6 L and a dose of about 1905 L/ha. It is noteworthy that fenoxycarb was applied mixed with pyriproxyfen. The temperature was 28 °C and the relative humidity equaled 51%. The PHI was considered to be 21 days. Fruit samples were collected two hours after application and then after 2, 7, 14, and 21 days (GAP). At the PHI, the same dose was applied again (CAP). The temperature was 26 °C and the relative humidity 44%. Two hours after the treatment samples were taken for residue analysis. The levels of pyriproxyfen in peach samples after 2 h and after 2, 7, 14, and 21 days were equal to 0.073 ± 0.005 mg/kg, 0.205 ± 0.018 mg/kg, 0.131 ± 0.008 mg/kg, 0.062 ± 0.006 mg/kg, and 0.05 ± 0.003 mg/kg, respectively (LOQ = 0.05 mg/kg). A half-life of 15.5 days was estimated. It is worthy to note that a t_1/2_ value of 5 days was found with fenoxycarb. In the pesticide treatment under CAP conditions, the sample harvested after 2 h had a residue level in pyriproxyfen of 0.123 mg/kg.

The same methodology was applied by Paya et al. [44] to grapes (*Vitis vinifera* var. Monastrell). The dose applied of Atominal (10% EC pyriproxyfen) was 0.06%. The temperature was 23°C and the humidity was 55%. The PHI was equal to 28 days. At that date, the same dose was applied to simulate a CAP situation. The temperature was 24 °C and the relative humidity 45%. The residues in pyriproxyfen at 2 h, 21 days, and 28 days were equal to 0.15, 0.06, and 0.06 mg/kg (LOQ = 0.05 mg/kg). After 2 h under CAP conditions, the concentration in pyriproxyfen was equal to 0.18 mg/kg.

Du et al. [45] studied the behavior of pyriproxyfen (10% SC) in *Pleurotus ostreatus* (oyster mushroom) and *Lentinula edodes* (shiitake mushroom) growing on two different substrates and using two application methods. For *P. ostreatus*, the substrate either included 50% corncob, 31% cottonseed, 17% bran, 1% gypsum, and 1% calcium hydroxide with a pH = 7.62 (S1) or 88% ground corncob, 10% wheat bran, 1% superphosphate, and 0.3% urea with a pH = 7.86 (S2). For *L. edodes*, the substrate either included 80% sawdust, 20% bran, and 1% gypsum with a pH = 6.52 (S3) or 87% sawdust, 13% wheat bran, and 0.1% gypsum powder with a pH = 6.73 (S4). Substrates were soaked with tap water, drained, sterilized in autoclave and cooled to room temperature before inoculation. In a first experiment, pyriproxyfen was mixed with the substrates at 56 mg/kg (T1 = recommended dosage (RD)), 167 mg/kg (T3 = 3 RD) and 504 mg/kg (T9 = 9 RD). The substrates were collected randomly from the sampling plots 2 h after being mixed with the insecticide to calculate the loss rate by sterilization. As regards *P. ostreatus* it was of 2.14%, 2.32%, 4.29%, 4.70%, 1.97%, and 3.86% in S1T1, S1T3, S1T9, S2T1, S2T3, and S2T9, respectively. For *L. edodes* it was equal to 10.5%, 8.57%, 3.72%, 12.7%, 9.53%, and 1.18% in S3T1, S3T3, S3T9, S4T1, S4T3, S4T9, respectively. The loss rate changed with the type of substrate and it was generally low. In all cases, it was the lowest of the pesticides tested by Du et al. [45] under the same conditions. Indeed, it is worth noting that β-cypermethrin, avermectin, and diflubenzuron were also tested on the S1 to S4 substrates with T1, T3, and T9 doses in relation with their recommended doses of application. For each combination experienced (i.e., S1T1 to S4T9) the loss rate of pyriproxyfen was much lower than those of the other pesticides.

To investigate the residue behavior of the pesticide by spraying, 1.5 times the recommended dosage (T1.5) was applied. The dosage of pyriproxyfen 10% SC was 750 g for 1 million mushroom sticks with one time spray. With both mushroom species and substrates, a strong decrease was rapidly observed in the substrates and at day 35 only few percentages of the initial concentration were found.

Analysis of the residues at the harvest time was also investigated. Pyriproxyfen was either mixed with the substrates or sprayed (twice) with 500 g and 750 g for 1 million mushroom sticks. Samples were collected at 1, 3, 5, 7, and 14 days. In the former situation, pyriproxyfen was not detected in both mushroom species. In the spraying experiments with *P. ostreatus*, the residues found at day 1 after the first spraying for S1T1 and S1T1.5 equaled 0.392 mg/kg and 0.364 mg/kg, respectively. After the second spraying, at day 1, 0.446 mg/kg and 4.23 mg/kg were found, respectively. From 3 to 14 days, <0.1 mg/kg was always recorded. For S2T1 and S2T1.5 at day 1 and after the first spraying, 0.586 mg/kg and 0.917 mg/kg were respectively found while for the other sampling times, <0.1 mg/kg was always detected. At the second spraying, the residues found in S2T1 and S2T1.5 samples equaled 1.75 mg/kg and 4.84 mg/kg at day 1 and 0.447 mg/kg and 0.611 mg/kg at day 3, respectively. Then, <0.1 mg/kg was always found for the other sampling times. Broadly the same trends were observed with *L. edodes* for S3T1 and S3T1.5. On contrary, the persistence was longer in S4 after the second spraying. After the first spraying, the residues found at day 1 equaled 0.545 mg/kg and 0.811 mg/kg for S4T1 and S4T1.5, respectively. The residues equaled 0.233 mg/kg and 0.695 mg/kg at day 3 and then always <0.1 mg/kg. However, regarding the second spraying, with T1, the residues found at 1, 3, 5, 7, and 14 days equaled 2.32, 1.41, 0.372, 0.344, <0.1 mg/kg, respectively. With T1.5, 3.82, 1.37, 0.495, 0.14, and <0.1 mg/kg were found respectively [45].

A similar study was performed by Du et al. [46] with button mushrooms (*Agaricus bisporus*) growing on a substrate including wheat straw (78%), chicken manure (20%) and slaked lime (2%). In the substrate, the residue of pyriproxyfen (10% SC) was <0.1 mg/kg three days after application. No residues were detected in the mushrooms when pyriproxyfen was mixed with the substrate. When pyriproxyfen was sprayed after few fruit bodies appeared and the button mushrooms were collected after the spraying, <0.001 mg/kg was found.

### 3.3. Residues in Fruits and Vegetables

The purpose of pesticide monitoring programs is to ensure that the fruits and vegetables available for consumption respect the MRLs allowed by the governmental authorities, no misuse of pesticides could result in unexpected residue levels and that the GAPs are maintained. More generally, there is a growing social desire to know the level of contamination of fruits and vegetables by pesticides. Thus, the results of the monitoring programs conducted by the Danish Veterinary and Food Administration from 2004 to 2011 [47] showed that on the 4936 samples of fruits and vegetables analyzed from various geographical origins, pyriproxyfen was only detected in 93 samples (1.88%). Pyriproxyfen was detected in eggplants (6.25%), grapefruits (8.39%), lemons (27.85%), mandarins and clementines (10.63%), oranges (4.05%), peppers, sweet (1.18%), pomelos (2.94%), and tomatoes (7.69%). It is worthy to note that on the 927 samples of cereals analyzed, pyriproxyfen was never detected [47]. Forty samples of parsley (*Petroselinum crispum* var. neapolitanum), 40 samples of spinach (*Spinacia oleracea*) and 40 samples of lettuce (*Lactuca sativa* var. longifolia) harvested and commercialized in farmer markets of the Hatay province (Turkey) were analyzed by liquid chromatography-tandem mass spectrometry (LC-MS/MS) for detecting the potential presence of 80 pesticides including pyriproxyfen [48]. All the parsley samples were contaminated by carbaryl, carbendazim, cymoxanil, cypermethrin, dichlorvos, ethiofencarb, metalaxyl, pirimiphos-methyl, simazine, and trichlorfon. Among the 25 pesticides detected in the parsley samples, the highest concentrations were found for carbendazim (0.111 mg/kg) and pendimethalin (0.289 mg/kg). Atrazine, carbendazim, cymoxanil, dichlorvos, ethiofencarb, metalaxyl, monolinuron, pirimiphos-methyl, simazine, and trichlorfon were found in all the lettuce samples. Among the 26 pesticides detected in the lettuce samples, the highest concentrations were recorded for acetamiprid (0.104 mg/kg), imidacloprid (1.22 mg/kg), and pendimethalin (0.354 mg/kg). Acetamiprid, dichlorovos, and pirimiphos-methyl were found in all the spinach samples. Among the 13 pesticides detected in these samples, carbendazim was found with concentrations ranging from 0.003 to 1.84 mg/kg and cymoxanil with concentrations ranging from <0.001 to 0.161 mg/kg. MRLs were exceeded in 70%, 45%, and 100% of the parsley, lettuce, and spinach samples, respectively. According to Esturk et al. [48] the farmers in Hatay were unconscious as regards the use of pesticides or showed a tendency to overdose them. Nevertheless, it is interesting to stress that despite these bad agricultural practices, pyriproxyfen was never detected (limit of detection (LOD) = 0.005 mg/kg) [48]. In 2014, 170 samples of 13 different fruits and 147 samples of 17 different vegetables were obtained from production farms located in the central and eastern region of Poland [49]. They were analyzed for the potential presence of 207 pesticides including pyriproxyfen. Pesticides were detected in 38.2% of the fruit samples and 16.3% of the vegetable samples. Pesticides were most often found in the samples of gooseberry (100%), apple (71.4%), blueberry (66.7%), currant (60.0%), and raspberry (55.1%). Among the vegetables pesticides were mainly found in the samples of tomato (50.0%), broccoli (50.0%), parsley root (21.4%), cucumber (16.7%), and Peking cabbage (11.8%). Violations of MLRs were found in two samples of raspberry (0.6%) and they concerned flutriafol, penconazole, and spirodiclofen. Pyriproxyfen was never detected (limit of quantification (LOQ) = 0.02 mg/kg). It is interesting to note that fenoxycarb (LOQ = 0.05 mg/kg) was also not detected in the different samples [49]. The same strategy was applied to 547 samples of 13 different fruits and 479 samples of 26 different vegetables originated from the region of south-eastern Poland. Again pyriproxyfen was not found [50]. Ten different vegetable commodities from the Asir region (Saudi Arabia) were evaluated for their pesticide contamination [51]. In this context, 211 samples were collected from supermarkets in which 80 pesticides (including pyriproxyfen) were analyzed. On the 26 tomato samples analyzed, pyriproxyfen was found four times with concentrations ranging from 0.033 to 0.167 mg/kg. On the 28 chili pepper samples analyzed pyriproxyfen was detected twice with concentrations of 0.043 and 0.056 mg/kg. On the 22 potato samples analyzed, pyriproxyfen was also found twice with concentrations of 0.013 and 0.026 mg/kg. Unfortunately, the LOD and LOQ values were not specifically given for pyriproxyfen [51]. Two hundred date (*Phoenix dactylifera* var Sukkari) samples (1–2 kg each) were collected from large markets in the Al-Qassim region in Saudi Arabia during 2016 to evaluate their potential contamination in pesticides [52]. Forty-two pesticides were analyzed including pyriproxyfen. Carbofuran, imidacloprid, acetamiprid, difenoconazole, carbendazim, metalaxyl, hexythiazox, oxadiazon, malathion, and indoxacarb were found in 4.5%, 4%, 3%, 3%, 2.5%, 2%, 1.5%, 1%, 0.5%, and 0.5% of the samples, respectively. The corresponding mean concentrations found were equal to 3.7 ± 1.4 µg/kg, 33 ± 16 µg/kg, 9 ± 6 µg/kg, 79 µg/kg, 226 ± 6 µg/kg, 30 ± 10 µg/kg, 133 ± 29 µg/kg, 289 ± 91 µg/kg, 136 µg/kg, and 114 µg/kg, respectively, pyriproxyfen was never detected (LOD = 0.22 µg/kg and LOQ = 0.73 µg/kg) [52].

The above examples show that whatever the type of sample and the agricultural practices used, pyriproxyfen is generally not detected in fruits and vegetables available for consumption. However, it is important to note that the analysis of its main metabolites was never made.

## 4. Concluding Remarks

In soils, the photodegradation of pyriproxyfen proceeds slowly with half-lives ranging from 10 to 20 weeks depending on the soil type and abiotic factors. Under artificial conditions, photodegradation is quicker, PYPAC and 4′-OH-Pyr being the main degradation products identified.

Whatever the type of soil, aerobic degradation of pyriproxyfen is fast. It is done in two steps, the former being quicker than the latter. A rather high variability can be found between the results of different studies even if the types of soils and the abiotic conditions are rather similar. Moisture, content in organic matter, presence of other pesticides or fertilizers, applied dose, and number of treatments also highly influence the degradation behavior of pyriproxyfen. 4′-OH-Pyr and PYPAC are main degradation products.

With low water solubility and high log Kow values, pyriproxyfen is adsorbed onto the soil surfaces and it is not a leacher. 4′-OH-Pyr being slightly less hydrophobic than pyriproxyfen, it is a little bit more mobile. On contrary, with a high solubility in water and low log kow value, PYPAC is highly mobile in the soils and it shows a high potential to leach to groundwater.

The evaluation of the fate of pyriproxyfen in soil allows us to better understand and estimate the potential risk posed by the insecticide and its degradation products to the soil biota. Thus, for example, although pyriproxyfen was found to have a lack of acute toxicity against adult earthworms (*Eisenia foetida*), this was not the case of its degradation products. Indeed, while the 50% lethal concentration in 72 h (72 h LC_50_) of pyriproxyfen equaled 672.29 µg/cm^2^, the 72 h LC_50_ values of 4′-OH-Pyr, DPH-Pyr, POP, POPA, PYPA, and PYPAC against earthworms equaled 30.39, 10.25, 13.29, 24.33, >75.52, and >78.79 µg/cm^2^, respectively [30]. In the same way, Ahemad [53] studied the potential adverse effects of pyriproxyfen (98%) on different plants. The insecticide showed the highest toxicity on the root and shoot dry biomass, leghaemoglobin chlorophyll content and seed protein in chickpea (*Cicer arietinum* L.); the nodule numbers in pea (*Pisum sativum*); the shoot N and root P in greengram (*Vigna radiata* L. Wiclzeck); and the nodule biomass, root N, root P, shoot P and seed yield in lentil (*Lens esculentus*). In general, the most adverse effects were observed on the growth parameters of chickpea and lentil. Coskun et al. [54] showed that the germination and seedling growth of maize (*Zea mays* L. saccharata Sturt.), the number of stomata and the leaf pigment contents depended on the pyriproxyfen concentrations in the culture medium. Impacts on the soil microbial communities have been also observed [55,56].

Applied to crops, pyriproxyfen is predominantly found at the plant surface and also it remains very often unchanged in some plant tissues. Minor metabolites are formed by hydroxylation at the 4′-position of the phenoxy ring or cleavage of ether linkages. Depending on the crop and the experimental conditions, the half-life of pyriproxyfen in plants is ranged from less than one week to about three weeks. The mode of application on the crops highly influences the fate of pyriproxyfen in plants as well as its potential toxicity against non-target organisms. The main formulations of pyriproxyfen are applied to crops by spraying. Insecticide spray drift highly contributes to the unwanted toxicity and it has been shown that the air-blast spraying produced more drift that gun spraying [57,58]. When sprayed to plants, pyriproxyfen behaves as a translaminar also named “local systemic” insecticide. This means that after absorption of the insecticide by one side of the leaf surface, a reservoir is formed within the leaf tissues providing a residual activity against foliar-feeding pests. Numerous abiotic and biotic factors influence leaf penetration of pyriproxyfen. In all cases, if the pyriproxyfen sprayed on the leaves reaches the target insects, it can also adversely impact the non-target insects found on these leaves. Although the subject is rather well-documented including the study of the adverse effects of pyriproxyfen against the predators of pests [59], parasitoids [60], and beneficial insects [61], there is a need for investigating on the long-term effects of sublethal concentrations of pyriproxyfen at the population level under field or semi-field conditions [62,63].

## Figures and Tables

**Table 1:** Half-life values (t_1/2_) of pyriproxyfen enantiomers in different types of soils and sand under aerobic conditions and with various experimental conditions (adapted from [27]).

Medium	Sand %	Silt %	Clay %	OM ^1^ %	MO ^2^ %	pH	t_1/2_ (+)-Pyr days	t_1/2_ (-)-Pyr days	Rq. ^3^
Sandy clay loam	63.2	12	24.8	7.57	10	8.49	22.28	23.02	
	63.2	12	24.8	7.57	25	8.49	16.7	16.12	
	63.2	12	24.8	7.57	25	8.49	14.23	13.40	1
	63.2	12	24.8	7.57	25	8.49	15.79	15.57	2
	63.2	12	24.8	7.57	50	8.49	25.29	25.20	
Clay loam	37.2	24	38.8	36.76	10	7.77	15.89	15.71	
	37.2	24	38.8	36.76	25	7.77	4.77	4.66	
	37.2	24	38.8	36.76	25	7.77	4.43	4.43	1
	37.2	24	38.8	36.76	25	7.77	7.80	7.67	2
	37.2	24	38.8	36.76	50	7.77	27.07	26.45	
Loam	32	44	24	20.48	25	6.55	7.01	7.10	
	32	44	24	20.48	25	6.55	6.48	6.42	1
	32	44	24	20.48	25	6.55	31.08	29.87	2
Sandy loam	73.2	16	10.8	28.68	25	8.17	4.82	5.62	
	73.2	16	10.8	28.68	25	8.17	4.38	4.98	1
	73.2	16	10.8	28.68	25	8.17	5.14	5.05	2
	73.2	16	10.8	28.68	25	8.17	11.99	14.68	3
Clay loam	26	38	36	6.05	25	5.18	9.96	9.64	
Sand	100	0	0	6.02	25	8.33	25.86	40.29	

^1^ OM = organic matter content, ^2^ MO = moisture. ^3^ Rq. 1 = pyriproxyfen and imidacloprid, 2 = pyriproxyfen and emamectin benzoate, 3 = pyriproxyfen applied twice.

**Table 2:** Half-life values (t_1/2_ in days) of pyriproxyfen enantiomers and those of its main metabolite in five types of soils under aerobic conditions (adapted from [30]).

Soil Type	pH	OM ^1^	SP1 ^2^	SP2	SP3	t_1/2_ (-)-Pyr	t_1/2_ (+)-Pyr	t_1/2_ (-)-4′-OH-Pyr	t_1/2_ (+)-4′-OH-Pyr
Loamy sand	8.10	12.4	845	85	70	2.11	2.11	3.29	2.80
Clay #1	8.05	47.9	281	130	589	8.39	6.14	5.11	6.56
Sandy clay loam #1	8	17.8	475	247	278	4.49	3.67	7.38	3.90
Clay #2	7.89	8.69	309	253	438	9.61	9.69	8.76	9.97
Sandy clay loam #2	7.94	8.31	642	155	203	7.31	8.75	11.21	13.30

^1^ OM = organic matter content in g/kg, ^2^ SP = soil particle size: (1) 2–0.05 mm, (2) 0.05–0.002 mm, (3) <0.002 mm.

**Table 3:** Koc values of pyriproxyfen in different soils and sand (adapted from [21]).

Medium	Sand %	Silt %	Clay %	OM ^1^ %	pH	CEC ^2^	Koc
Loam	56	30	15	8.2	7.1	32	1.30 × 10^4^
Clay loam	55	26	19	1.9	7	6.3	5.80 × 10^4^
	21	47	32	5	7	21	1.10 × 10^4^
Sandy loam	72	18	11	0.9	7.2	2.8	2.70 × 10^4^
	60	25	15	1.65	8	9.7	1.26 × 10^4^
Silt loam	29	58	13	1.1	7	13	2.69 × 10^4^
Silty clay loam	7	53	49	1.4	7.8	27	3.42 × 10^4^
Sand	97	1	2	0.3	5.4	1.1	1.16 × 10^4^

^1^ OM = organic matter content, ^2^ CEC = cation exchange capacity in meq/100 g.

**Table 4:** Koc values of 4′-OH-Pyr and PYPAC in different soils and sand (adapted from [21]).

Medium	Sand %	Silt %	Clay %	OM ^1^ %	pH	CEC ^2^	Koc 4′-OH-Pyr	Koc PYPAC
Sand	92	3.6	4.4	0.22	6	0.82	4250	85
Sandy loam	75	18	7.2	0.96	6.9	6.6	3810	21
Silt loam	35	54	11	1.8	6.9	8.9	3060	32
Clay loam	33	28	39	2.1	7.9	15.8	920	9

^1^ OM = organic matter content, ^2^ CEC = cation exchange capacity in meq/100 g.
